# Astrogliosis in multiple sclerosis and neuro-inflammation: what role for the notch pathway?

**DOI:** 10.3389/fimmu.2023.1254586

**Published:** 2023-10-23

**Authors:** Pierre Mora, Candice Chapouly

**Affiliations:** Université de Bordeaux, Institut national de la santé et de la recherche médicale (INSERM), Biology of Cardiovascular Diseases, Pessac, France

**Keywords:** multiple sclerosis, astrogliosis, NOTCH signaling, neuro-inflammation, astrocytes

## Abstract

Multiple sclerosis is an autoimmune inflammatory disease of the central nervous system leading to neurodegeneration. It affects 2.3 million people worldwide, generally younger than 50. There is no known cure for the disease, and current treatment options - mainly immunotherapies to limit disease progression - are few and associated with serious side effects. In multiple sclerosis, disruption of the blood-brain barrier is an early event in the pathogenesis of lesions, predisposing to edema, excito-toxicity and inflammatory infiltration into the central nervous system. Recently, the vision of the blood brain barrier structure and integrity has changed and include contributions from all components of the neurovascular unit, among which astrocytes. During neuro-inflammation, astrocytes become reactive. They undergo morphological and molecular changes named “astrogliosis” driving the conversion from acute inflammatory injury to a chronic neurodegenerative state. Astrogliosis mechanisms are minimally explored despite their significance in regulating the autoimmune response during multiple sclerosis. Therefore, in this review, we take stock of the state of knowledge regarding astrogliosis in neuro-inflammation and highlight the central role of NOTCH signaling in the process of astrocyte reactivity. Indeed, a very detailed nomenclature published in nature neurosciences in 2021, listing all the reactive astrocyte markers fully identified in the literature, doesn’t cover the NOTCH signaling. Hence, we discuss evidence supporting NOTCH1 receptor as a central regulator of astrogliosis in the pathophysiology of neuro-inflammation, notably multiple sclerosis, in human and experimental models.

## 1 Introduction

Neurodegeneration, defined as neuronal cell loss, is the major cause underlying behavioral and psychological abnormalities, notably in multiple sclerosis (MS). It represents a major public health issue as life expectancy increases. One common feature of neurodegeneration is vascular impairment: the Central Nervous System (CNS) vasculature, through its regulatory mechanisms including solute transporters, receptor-mediated transcytosis and low levels of immune receptors, represents a physical blood brain barrier (BBB) which separates blood components from the brain parenchyma (1). However, during neuropathology, the BBB becomes permeable leading to parenchymal inflammatory infiltration (2). Increased BBB leakage is a common feature of several neurological conditions such as dementia, ischemic brain injury or neuro-inflammatory disorders like MS with a strong detrimental effect.

Importantly, a wealth of literature has recently enabled a change in the vision of the BBB structure and integrity which has expanded to include contributions from all components of the neurovascular unit (NVU), among which endothelial cells, pericytes, microglia and astrocyte endfeet (glia limitans) (3). Strikingly, our group recently published a paper highlighting the capacity for bidirectional signaling between endothelial cells and astrocytes from the NVU (4); however, how these signals participate to cerebrovascular impairment, notably BBB dysfunction and neuro-inflammation remains unclear and is of considerable translational interest to the field of neuro-immunology. Specifically, while it is now established that BBB breakdown leads to inflammatory infiltration into the perivascular space during neuropathology, the role of astrocytes from the glia limitans appears trickier. Indeed, astrocytes, described as reactive, are emerging as “Dr Jekyll and Mister Hyde” cells, having complex roles in both recruiting and restricting neuro-inflammatory infiltration. Reactive astrocyte behavior is determined in a context-specific manner by signaling events that vary with the nature and severity of CNS insults. Indeed, during infections (HIV, Herpes virus), and in Alzheimer’s disease, Parkinson’s disease and MS, reactive astrocytes have been shown, on one hand, to produce pro-inflammatory and pro-permeability factors and, on the other hand, to produce neuroprotective factors (5, 6).

A better understanding of the molecular pathways responsible for the production of these beneficial or deleterious factors could enable the development of new therapies targeting astrocytes in MS in which currents treatments are mostly directed against the immune system and responsible for many side effects.

Among the pathways involved in controlling astrocyte reactivity, notch receptor 1 (NOTCH1) has been identified as a central effector of astrogliosis in a wide range of neuropathological contexts. The NOTCH signaling, which is highly conserved in vertebrates, is stimulated by the interaction of NOTCH receptor with its ligands, Delta (DLL) and Jagged (JAG), which are trans-membrane proteins with large extracellular domains. The precise number of notch receptors differ between species (7): in vertebrates, 4 different notch receptors have been identified and in the NVU, NOTCH1 is expressed by astrocytes, neurons and endothelial cells, notch receptor 4 (NOTCH4) by endothelial cells (8–10) and notch receptor 3 (NOTCH3) by mural cells, or pericytes (11, 12).

The NOTCH pathway is expressed in neural stem cells during development and is involved in neuron – glia cell fate decision. Strikingly, NOTCH1 signal transduction is reactivated in reactive astrocyte populations after intra-cerebral hemorrhage (13), in stroke (14) and in several neuro-inflammatory conditions such as amyotrophic lateral sclerosis (ALS) (15), MS and experimental auto-immune encephalomyelitis (EAE) (16, 17).

The targeting of NOTCH in therapeutics was first studied in oncology. More recently, the pathological role of the NOTCH pathway in the control of inflammation has been demonstrated in numerous autoimmune diseases, and is currently being tested as a therapeutic target in allergic asthma and graft-versus-host disease. Numerous strategies have been tested and are still under development to inhibit this pathway. However, non-specific inhibition of the NOTCH pathway has failed due to an unfavorable safety profile. A better understanding of the ligand-receptor interactions and their effects during neuro-inflammation is therefore necessary to determine their therapeutic interest and identify more specific targets of the pathway.

This review will address the role of reactive astrocytes in controlling BBB homeostasis and inflammatory infiltration of the CNS, under pathological conditions, with a focus on MS. More specifically, we will address the involvement of the NOTCH signaling in the control of astrogliosis, to provide a comprehensive review of what is known about this pathway and the prospects for further work that it offers.

## 2 Subsections relevant for the subject

### 2.1 Multiple sclerosis

MS is an inflammatory autoimmune disease of the CNS, causing motor, sensory, cognitive, visual and sphincter disturbances. It is a progressive neurological disease affecting young adults, with varied and sometimes inconspicuous or non-specific signs and symptoms.

#### 2.1.1 The first anatomo-pathological observations in humans

The earliest evocative description of MS date back to the 14th century, with Lidwine de Schiedam, holly patroness of ice skating, who suffered from a slowly progressing disease with periods of recovery, and whose symptomatology consisted of gait disturbance, paralysis of the right upper limb, unilateral loss of sight, stabbing facial pain and trouble swallowing (18).

**Figure 1:**
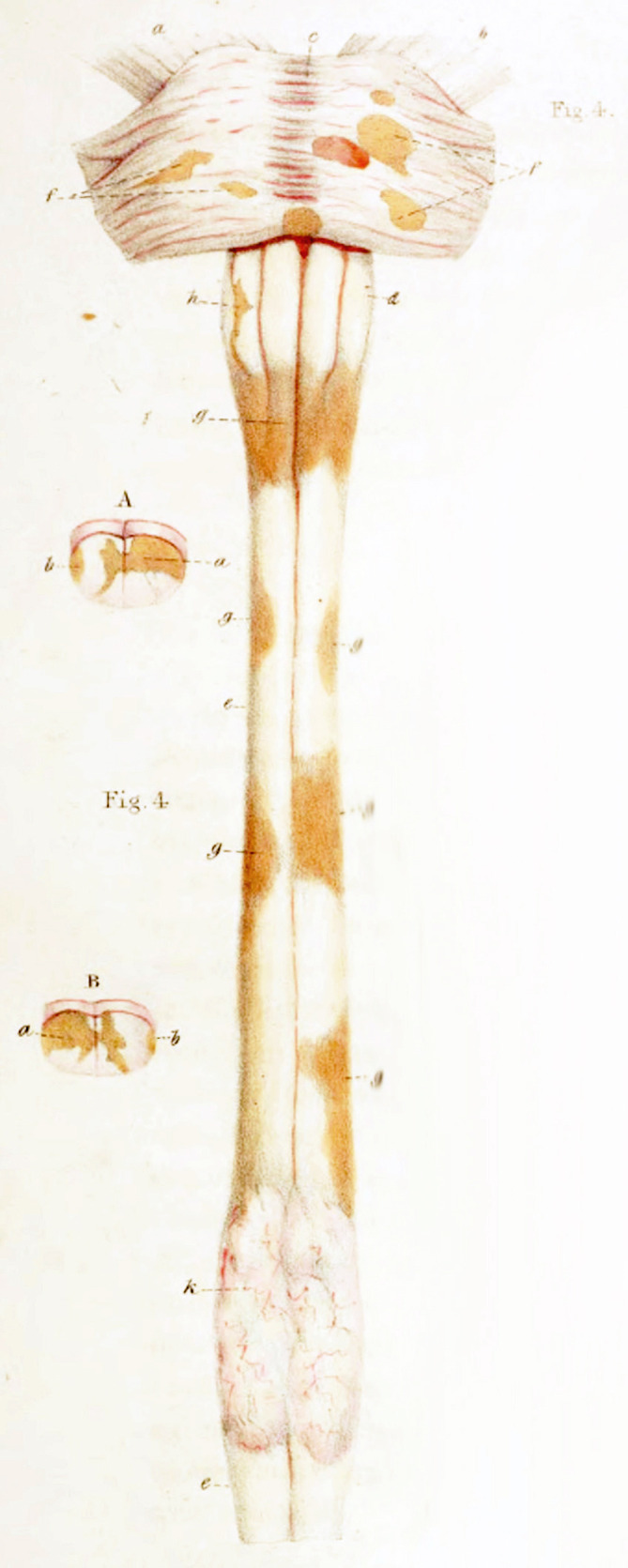
Illustration of spinal sclerotic lesions by Sir Robert Carswell.

In the first half of the 19th century, anatomo-pathological atlases by Jean Cruveilhier (19) and Sir Robert Carswell (20) focused on the spinal cord, with illustrations of spinal sclerotic lesions (**Figure 1** Illustration of spinal sclerotic lesions by Sir Robert Carswell).

However, it was not until 1868 that Jean-Marie Charcot’s anatomical-clinical approach made the connection between these post-mortem histological observations of medullary sclerotic lesions and the clinical signs that had been described for centuries. He described the pathogenesis of the disease, demyelination and glial proliferation, and thus proposed the diagnostic criteria for the pathology he calls MS. These signs - dysarthria, nystagmus and intention tremor - became known as the Charcot’s triad (21).

#### 2.1.2 Multiple sclerosis epidemiology

Several epidemiological studies have highlighted an increase in MS incidence in the order of 15% since 2013, bringing the average incidence worldwide in 2020 to 43.95 per 100,000 people (95% confidence interval [43.90; 44.01]), with a strong loco-regional disparity, with European and American countries being more affected than Asian or African countries (22).

MS is affecting young adults and is twice as common in women as in men (23).

One of the major limitations of epidemiological studies is that they are based solely on self-reporting of incidence and prevalence. The variable reliability of databases around the world, therefore, partly explains the differences observed.

**Figure 2:**
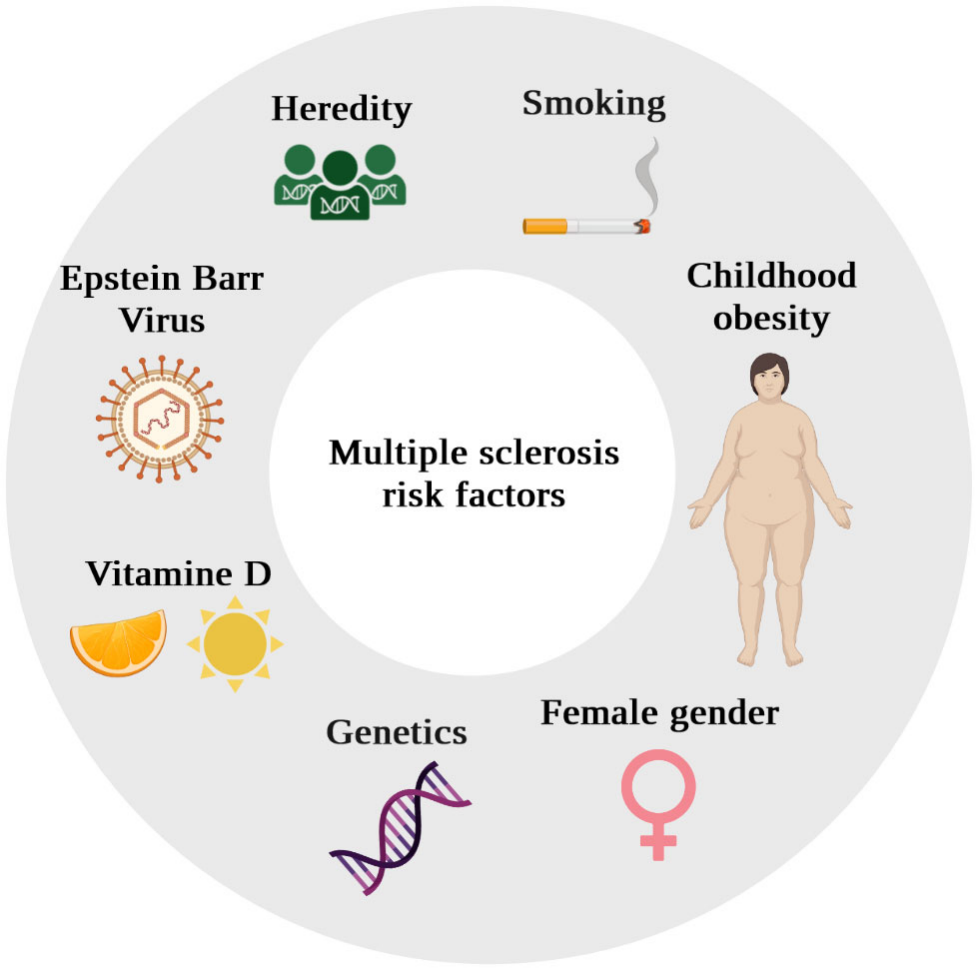
Schematic diagram of multiple sclerosis (MS) risks factors.

Beyond the declarative performance of different national health systems, these geographical epidemiological differences reflect the existence of genetic predisposing factors and environmental risk factors (**Figure 2**).

#### 2.1.3 The numerous forms of multiple sclerosis

The positive diagnosis of MS is based on clinical and para-clinical arguments. In order to standardize this diagnosis, the McDonald criteria were developed by the International Panel on Diagnosis of Multiple Sclerosis, comprising European and American neurologists. These criteria are based on the spatial and temporal dissemination of lesions (24).

Despite these common diagnostic criteria, the different clinical presentations of MS are extremely heterogeneous.

As early as 1996, an American-European consensus, The International Advisory Committee on Clinical Trials in Multiple Sclerosis, classified the different clinical course of MS into four categories:

- Relapsing-remitting MS (RR-MS)
- Secondarily progressive MS (SP-MS)
- Primary-progressive (PP-MS)
- Progressive-relapsing (PR-MS)

RR-MS is characterized by alternating attacks and periods of complete recovery or incomplete recovery with minor residual deficit and by a lack of disease progression between relapses. Attacks develop gradually, over several days. It is the most frequently diagnosed form, accounting for 85% of cases. RR-MS patients may go on to develop SP-MS, corresponding to a continuous progression of symptoms. PP-MS corresponds to a continuous and progressive deterioration of neurological functions, with no clear recovery periods, but with potential plateau phases or minor improvement. It affects 10-15% of patients at diagnosis, and is more common in men. The average age at diagnosis is also higher (40 versus 30 for RR forms). PR-MS, which is less common, corresponds to a primary progressive form with episodes of acute relapses, with or without periods of recovery (25).

In 2013, an update of these recommendations introduced a new syndrome, the clinically isolated syndrome, and the authors proposed two concepts for characterizing the disease: disease activity and disease progression (26). The clinically isolated syndrome (CIS) corresponds to the first clinical presentation of an episode possessing the characteristic signs of a demyelinating disease, without fulfilling all of Mac Donald’s criteria for diagnosis, notably the notion of temporal dissemination (27).

Activity can be assessed clinically (clinical signs of relapse) or by imaging. Progression is determined on the basis of clinical evidence of worsening disability.

The new phenotypes proposed are as follows (**Table 1** Evolution of clinical courses description of relapsing multiple sclerosis and **Table 2** Evolution of clinical courses description of progressive multiple sclerosis):

**Table 1:** Evolution of clinical courses description of relapsing multiple sclerosis.

Relapsing multiple sclerosis			
MS clinical courses as described in 1996 guidelines		MS clinical courses as described in 2013 guidelines	
RR-MS	Full recovery between relapses	CIS	Not active
	Incomplete recovery between relapses		Active
		RR-MS	Not active
			Active

**Table 2:** Evolution of clinical courses description of progressive multiple sclerosis.

Progressive multiple sclerosis			
MS clinical courses as described in 1996 guidelines		MS clinical courses as described in 2013 guidelines	
Progressive disease	PP-MS	Progressive disease (both PP-MS and SP-MS)	Active with progression
	SP-MS		Active without progression
	PR-MS		Not active with progression
			Not active without progression

- Relapsing disease:
  Active CIS
  Not active CIS
  Active RR-MS
  Not active RR-MS
- Progressive disease: PP-MS or SP-MS
  Active progressive disease with progression
  Active progressive disease without progression
  Progressive not active disease with progression
  Not active and without progression disease (stable disease)

Alongside progression, the notion of disease worsening was defined in 2020. Unlike progression, which corresponds to an accumulation of disability, independently of any relapses, during the progression phase, disease aggravation corresponds to an increase in disability, whether resulting from a residual relapse deficit or from progressive disability during the disease progression phase (28).

Disruption of the BBB, and more broadly of the NVU, comprising astrocytic endfeet or “glia limitans”, is an early and fundamental element in the pathogenesis of MS and plays an important part in the diagnosis of MS notably through medical imaging.

#### 2.1.4 Inflammatory infiltration at the neurovascular unit, a key stage in multiple sclerosis pathogenesis

##### 2.1.4.1 Definition of the neurovascular unit

The CNS has high energy consumption but a very low storage capacity. This massive energy consumption requires constant exchange between the blood and the brain. Thus, despite representing only 2% of the human body’s mass, the CNS receives over 20% of blood flow to satisfy its oxygen and nutrient requirements (29, 30). However, the CNS is a fragile tissue, with a low capacity for renewal, and therefore requires protection from microorganisms.

Under physiological conditions, the CNS is protected from endogenous and exogenous elements present in the systemic circulation by a physical barrier which, in vertebrates, is called the BBB. The BBB, a major evolutionary advantage, is uniquely structured to meet the specific metabolic needs of the parenchyma. The highly complex structure of the BBB is the subject of numerous studies; however there are still many grey areas on its structure and functions.

Originally, this barrier was described as predominantly endothelial in nature, in association with mural cells (pericytes or smooth muscle cells) and the basal matrix. However, this simplistic view of the BBB has long been outdated. The increasing complexity of the BBB, with the discovery of new cell types such as microglia and astrocytes within its structure, has led to the emergence of a new consensual concept named the NVU. The NVU is a cellular network that modulates blood flow in response to glial and neuronal energy demand, and regulates the passage of molecules and cells into the brain parenchyma. Its organization and architecture are scrupulously controlled in order to fulfill these missions.

##### 2.1.4.2 Mechanisms of neurovascular unit inflammatory infiltration in multiple sclerosis

The most widespread hypothesis to date, to explain onset of MS pathology, is the infiltration of auto-reactive lymphocytes into the CNS through the NVU. These auto-reactive cells are the consequence of a loss of tolerance to certain CNS self-proteins, notably myelin, following cross-reactivity after infection (31).

It is now commonly accepted that the NVU is composed of 2 barriers that leukocytes must cross sequentially: a first barrier, the BBB, composed of endothelial cells, and a second barrier, the glia limitans, composed of astrocyte endfeet. These two barriers delimit a zone known as the perivascular space (32, 33).

The passage of leukocytes across the BBB involves several stages: capture, rolling, activation, adhesion, crawling, and diapedesis. Diapedesis corresponds to the migration of leukocytes through the endothelium (34).

During the onset of MS disease, neuro-inflammation occurs and endothelial cells from the BBB become activated, participating in the migration of plasmatic proteins and leukocytes from the systemic circulation to the parenchyma. Activated endothelial cells undergo changes including increased expression of adhesion molecules, pro-inflammatory cytokines and chemokines, combined with reduced expression or disorganization of junctional molecules. Increased expression of the selectin family proteins, selectin P (SELP) and selectin E (SELE), on endothelial cells leads to increased leukocyte rolling via binding of selectin P ligand (SELPLG) located on T lymphocytes (35). However, this rolling mechanism is not absolutely necessary as, interestingly, selectins appear to be dispensable in the induction of EAE (36, 37).

T lymphocytes, to stop completely, then require G protein-coupled receptor-dependent signaling, notably atypical chemokine receptor 1 (ACKR1). This arrest step is controlled by integrins, integrin subunit alpha L (ITGAL) and integrin subunit alpha 4 (ITGA4), and their endothelial ligands, intercellular adhesion molecule 1 (ICAM1) and vascular cell adhesion molecule 1 (VCAM1) respectively (38, 39).

T lymphocytes then crawl out in search of a suitable site for diapedesis. Until the early 2000s, the commonly accepted hypothesis was that mononuclear cells cross the endothelial barrier via a paracellular passage phenomenon known as “paracellular diapedesis”, following a reorganization of tight junctions sometimes referred to as the “zipper model” (40, 41). Since then, numerous studies have demonstrated the existence of a transcellular passage known as “transcellular diapedesis”, which spares the tight junctions (42). Transcellular passage has been much less studied than paracellular passage, but it would appear that under neuro-inflammatory conditions, the endothelial passage of circulating blood cells is predominantly transcellular. This hypothesis is supported by the fact that overexpression of adhesion molecules, notably ICAM1, found in neuro-inflammatory conditions, favors transcellular passage. Furthermore, the decrease in junctional integrity in endothelial cells does not correlate with an increase in paracellular diapedesis but rather with an increase in transcellular diapedesis (43, 44).

In the perivascular space, CD4+ cells encounter their antigens via antigen-presenting cells and perivascular macrophages. This step acts as a final checkpoint before the passage of the second barrier, the glia limitans, with CD4+ T cells remaining sequestered in the perivascular space in its absence (45).

Lymphocyte passage through the glia limitans requires active recruitment and enzymatic cleavage of glial barrier components. In the perivascular space, macrophages produce the metallo-proteinases matrix metallopeptidase 2 (MMP2) and matrix metallopeptidase 9 (MMP9), which destabilize the glia limitans and allow leukocytes to pass into the parenchyma. Substrates for MMP2 and MMP9 include dystroglycan, which anchors astrocytic endfeet to the parenchymal basement membrane via a high-affinity interaction with laminin 1 and 2, perlecan and agrin (46, 47).

In this review, we will focus on astrocytes, key components of the NVU; precisely we will explore how astrocytes control BBB homeostasis and inflammatory infiltration under pathological condition such as MS and EAE.

### 2.2 Astrocytes as part of the neurovascular unit, central players of neuro-inflammation pathophysiology

Astrocytes represent one of the most abundant cell types in the CNS, accounting for between 20% and 50% of the total number of CNS cells depending on the species, and help maintain parenchymal homeostasis during development, in physiological conditions and during aging. Originally, astrocytes were considered solely as a structural support cell population for neurons, but since the early 2000s, numerous studies have demonstrated a much more diverse range of roles for astrocytes physiologically and under pathological conditions (48).

The morphological heterogeneity of astrocytes has long been described. Historically, a distinction was made between fibrous astrocytes in the white matter and protoplasmic astrocytes in the gray matter (49–51). Recent technological advances have made it possible to identify different astrocyte subtypes within these two populations. Protoplasmic astrocytes, located in the gray matter, are characterized by short, numerous extensions that enable them to come into contact with other astrocytes as well as other cell types such as microglia or neurons. In white matter, astrocytes are fibrous or fibrillary, with a smaller cell body. Their extensions are longer and fewer.

This morphological difference based on the anatomical location of astrocytes is not only due to differences based on the origin of these cells, but also to environmental stimuli. Indeed, when exogenous astrocytes are grafted, they acquire a protoplasmic or fibrous morphology depending on the area in which they are grafted.

#### 2.2.1 Astrogliosis, a response mechanism to injury

Astrocytes are quiescent cells which undergo profound morphological and functional changes in response to stress. The nature of this stress is variable: ischemic, cytokinic, traumatic or infectious, for example.

Numerous names exist to describe these phenomena, the most common and those used in the remainder of this manuscript being “astrogliosis” to describe all the changes observed, and “reactive astrocytes” to describe astrocytes that have undergone these changes.

#### 2.2.2 Reactive astrocyte behavior under neuro-inflammatory conditions

##### 2.2.2.1 Blood brain barrier homeostasis

**Figure 3:**
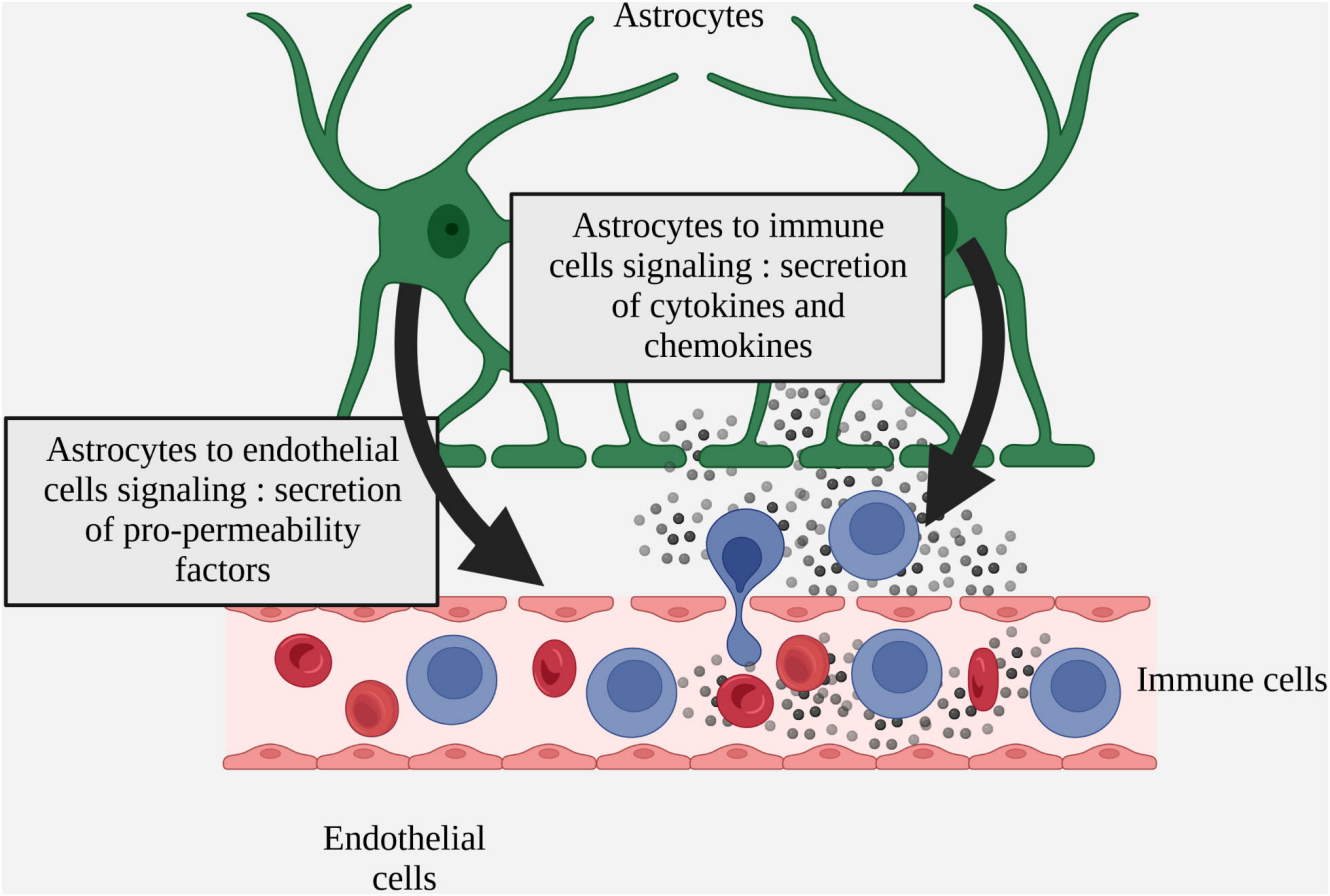
Simplified drawing of reactive astrocyte soluble signals to endothelial and inflammatory infiltrated cells.

In MS, several studies demonstrate both beneficial and deleterious effects of astrogliosis on BBB homeostasis via the secretion of factors by reactive astrocytes. In MS patients and more widely in various models of neuro-inflammation, reactive astrocytes have been identified as secreting factors that are either beneficial or deleterious to BBB integrity, notably by regulating the expression of endothelial junction proteins and endothelial activation markers (**Figure 3** Simplified drawing of reactive astrocyte soluble signals to endothelial and inflammatory infiltrated cells).

###### 2.2.2.1.1 Vascular endothelial growth factor A

The first reports of vascular endothelial growth factor A (VEGFA) expression by glial cells date back to the early 90s in human gliomas (52) in the process of retinal angiogenesis in rats or cats (53) or *in vitro* in rat astrocyte cultures subjected to hypoxic conditions (54). In 1999, MJ Merril’s laboratory demonstrated that chronic exposure of healthy rat brains to VEGFA, delivered by a pump or a viral vector, causes an increase in vascular permeability, measured by X-ray after intravenous administration (55). Still in rats, the authors observe VEGFA expression at day 6 post EAE induction and an increase in its production over time. VEGFA expression in the animals’ CNS correlates with parenchymal infiltration of cluster of differentiation 45 (CD45) + cells (56). In 2002, the same laboratory demonstrated astrocytic overexpression in cortical lesion areas of MS patients. GR John and AT Argaw’s team then demonstrated that astrocytic VEGFA expression is partly regulated by IL-1β. IL-1β induces HIF1α production via nuclear factor-kappa B (NFκB). HIF1α then enables the formation of a heterodimeric HIF1α/HIF1β complex which translocates to the nucleus and activates transcription of target genes including VEGFA (57). *In vitro*, adding VEGFA to human brain microvascular endothelial cells (hBMVEC) reduces the expression of the junction proteins claudin5 (CLDN5) and occludin (OCLN). *In vivo*, intra-cortical stereotactic administration of VEGFA to wild-type mice induces increased permeability and decreased expression of CLDN5 and OCLN (58). By creating a mouse model of astrocytic *VegfA* knockdown under *glial fibrillary acidic protein (Gfap)* promoter (*Vegfa^ACKO^* mice), the authors then showed that the clinical signs of EAE in these mice are extremely reduced, making the IL-1β/VEGFA/CLDN5 axis an interesting therapeutic target. Furthermore, downstream of VEGFA, the authors showed that the reduction in endothelial CLDN5 expression by astrocytic VEGFA is mediated by the activation of the endothelial vascular endothelial growth factor receptor 2 (VEGFR2), which activates the endothelial nitrite oxide synthase (eNOS) via the phospholipase C gamma (PLCγ). The use of an eNOS inhibitor, named Cavtratin, in EAE mice also results in a reduction in clinical signs of the condition, similar to the reduction observed in *Vegfa^ACKO^* mice (59).

Despite work in GR John’s laboratory on the importance of VEGFA in BBB opening under neuro-inflammatory conditions, VEGFA blockade does not completely abrogate the clinical signs of EAE, suggesting that other pathways are involved in BBB opening.

###### 2.2.2.1.2 Thymidine phoshorylase

By screening human astrocytes in culture stimulated by the pro-inflammatory cytokine IL-1β, Chapouly et al. identified the thymidine phoshorylase (TYMP) as a pro-permeability factor (6). The involvement of TYMP in tumor angiogenesis has previously been demonstrated in certain cancers of the digestive sphere (60–63), in breast cancer (64) and in lung cancer (65). The endothelial effect of TYMP is due not only to the mature protein itself, but also to one of its degradation products, 2-deoxy-D-ribose (DDR). TYMP catalyzes thymidine into thymine and 2-deoxy-D-ribose-1-phosphate, which is subsequently dephosphorylated to give DDR (66).

Chapouly et al. demonstrated that DDR has the same effects as VEGFA in reducing the expression of the junction proteins CLDN5 and OCLN both *in vitro* and *in vivo*. The authors also demonstrated that the effects of VEGFA and DDR are synergistic. However, despite these similar effects, sequencing of endothelial cells incubated with VEGFA and DDR showed that the genes modulated by these two factors are different, and of the 80 genes modulated by VEGFA and 41 modulated by DDR, only 9 are common (6).

###### 2.2.2.1.3 Sonic hedgehog and desert hedgehog

In mammals, there are 3 ligands of the hedgehog pathway: sonic hedgehog (SHH), indian hedgehog (IHH) and desert hedgehog (DHH). They are synthesized as precursors which undergo post-translational modifications: cleavage, C-terminal cholesterol modification and N-terminal palmitoylation. Hedgehog proteins initiate their signaling by binding to the patched receptor 1 (PTCH1), resulting in the removal of smoothened (SMO) inhibition. SMO then cleaves the *glioma-associated oncogene transcription factors (GLI)* which act as transcriptional effectors of the pathway (67). The critical role of the hedgehog signaling in neuro-inflammation was first highlighted in 2011 by A. Prat’s team; this study revealed that under MS or EAE conditions, the morphogen SHH is expressed by reactive astrocytes and participates in the maintenance of BBB homeostasis (68). However, following this discovery, our group found that, while SHH is expressed by activated astrocytes, DHH is physiologically expressed by endothelial cells in adults, particularly at the BBB (69). We demonstrated that endothelial DHH is down-regulated during EAE and that endothelial knockdown of *Dhh* is sufficient to induce BBB permeability by inhibiting tight junction expression. In parallel, our team has published a study demonstrating that ectopic expression of SHH by activated astrocytes under EAE condition induces BBB disruption by acting as a competitive antagonist of DHH at the endothelial level. Indeed, in a context of chronic neuro-inflammation, the two ligands compete at the level of their binding to the PTCH1 receptor on endothelial cells: thus, massive secretion of SHH by astrocytes contributes to the displacement of DHH from its receptor at the BBB, with consequent rupture of the NVU. This study has resolved some inconsistencies in the literature concerning the role of the hedgehog signaling in the NVU biology. Indeed, for a long time, the absence of specific inhibitors of each hedgehog ligand or of ligand-specific knockdown mice in a given cell type contributed to erroneous conclusions. The use of antibodies that do not discriminate between the three hedgehog ligands, as well as the use of SMO inhibitors and *Smo* knockdown mouse models, are the major causes of these wrong conclusions (69).

##### 2.2.2.2 Glial scar formation

After injury, necrotic nerve tissue is separated from healthy tissue by a glial scar composed mainly of proliferative astrocytes (70). Since the first observations of the glial scar in the early 20th century (71), quantitative and qualitative knowledge of its composition but also of its interactions with other cell types in the NVU has evolved considerably.

In 2013, MV Sofroniew’s laboratory highlighted the phenotypic heterogeneity of the astrocytes composing this scar in an acute model of spinal cord injury. In immediate contact with the lesion, proliferation is high and astrocytes will orient themselves parallel to the lesion via signal transducer and activator of transcription 3 (STAT3)-dependent signaling (72). This scarring protects viable neurons by segregating inflammatory cells in the lesion zone.

In models of chronic neuro-inflammation, inflammatory cell clusters have long been observed (73). In 2009, the same team demonstrated, in a mouse model of EAE, an organization of astrocytes similar to that found following spinal cord trauma. Indeed, reactive astrocytes in areas of EAE-induced inflammatory injury also form a scar that prevents the infiltration of inflammatory cells to the entire parenchyma (74).

In a more recent study, GR John’s team demonstrated the expression by reactive astrocytes of tight junction proteins usually found in tight epithelia such as the digestive or renal epithelia. The main tight junction proteins identified are claudin1 (CLDN1) and claudin4 (CLDN4). In an *in vitro* model of astrogliosis, a knockdown of these junction proteins leads to disorganization of reactive astrocyte networks and a defect in CD4+ T cell segregation in co-culture. *In vivo*, induction of EAE in an astrocytic *Cldn4*-deficient mouse model is associated with a milder pathology and less inflammatory infiltration of the parenchyma (75).

Subsequent work in EAE also showed that junctional adhesion molecule-A (JAM-A), in addition to its function as a tight junction, also plays a role in astrocyte-T cell communication and promotes the passage of the latter from the perivascular space into the parenchyma (76).

##### 2.2.2.3 Astrogliosis and inflammatory infiltration

**Figure 4:**
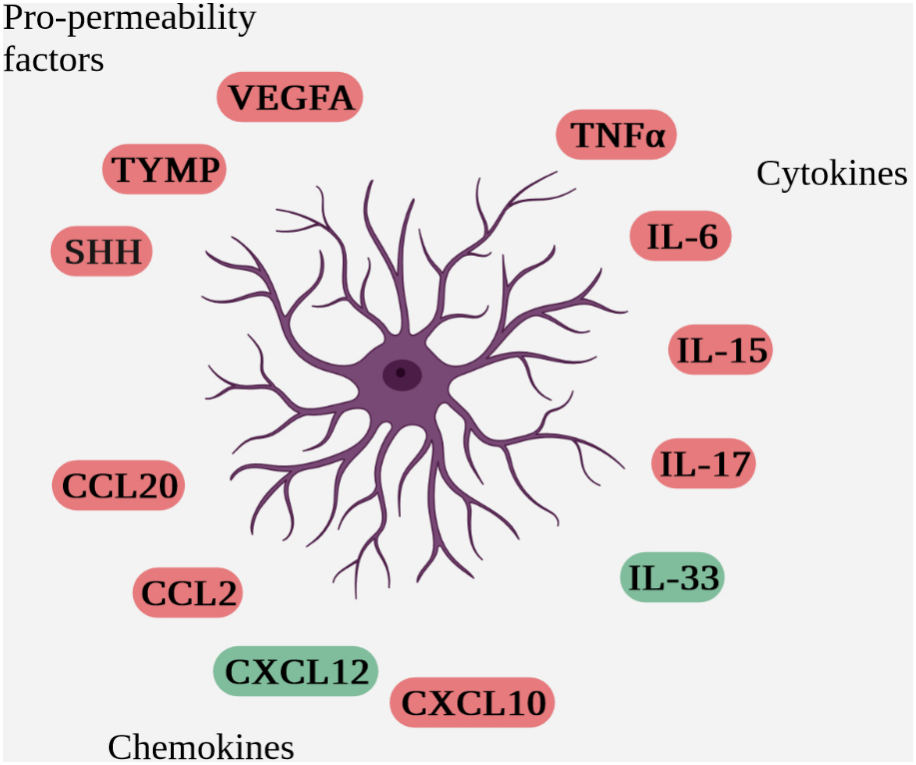
Schematic overview of soluble factors secreted by reactive astrocytes during neuro-inflammation (deleterious factors are in red and protective factors in green).

Within the NVU, astrocytes are heavily involved in communication with immune cells. In addition to direct contact between astrocytes and immune cells during passage through the parenchyma, their anatomical location in contact with vessels enables juxtacrine and paracrine communication via the secretion of soluble factors with immune cells trapped in the perivascular space (**Figure 4** Schematic overview of soluble factors secreted by reactive astrocytes during neuro-inflammation (deleterious factors are in red and protective factors in green)).

###### 2.2.2.3.1 Cytokines

Tumor necrosis factor-alpha (TNF-α) is overexpressed in acute and chronic active lesions in MS brains (77). However, this cytokine has been identified as having both beneficial and deleterious roles in EAE. This may be explained by the fact that TNF-α exists in two forms: a soluble form (solTNF) and a membrane-bound form (memTNF). Thus, in EAE-induced mice, using TNF-α inhibitors directed against the different forms, Brambilla et al. demonstrated that solTNF, by binding to tumor necrosis factor receptor 1 (TNFR1), is responsible for pro-apoptotic and pro-inflammatory actions and that memTNF, by binding to TNFR2, possesses anti-inflammatory and remyelinating actions (78). More recently, Plumb et al. demonstrated increased expression of ADAM metallopeptidase domain 17 (ADAM17), or TNF-α converting enzyme (TACE), in reactive astrocytes within active white matter lesions in MS patients. ADAM17 is an enzyme that cleaves soluble TNF-α, suggesting a neurotoxic role for astrocytic TNF-α (79). However, it should be noted that astrocytes express TNFR1 but also TNFR2 and that the anti-inflammatory and pro-myelinating role associated with astrocytic TNFR2 activation has been demonstrated in a CXCL2-dependent cuprizone model (80).

Interleukin-6 (IL-6) is notably overexpressed by activated astrocytes in the brains of MS patients at the level of acute and chronic active lesions (81, 82). Although several teams have focused on IL-6 under neuro-inflammatory condition, its exact role in the pathophysiology of MS is still unknown. Some studies suggest a beneficial role for astrocytic IL-6, notably because, in human brains, IL-6+ astrocytes are predominantly located close to healthy oligodendrocytes (82). Conversely, several studies in mice suggest a deleterious role for IL-6. In a mouse model with total *Il-6* knockdown, *Il-6*-deficient mice are completely resistant to EAE induction (83). In an inducible mouse model of *Il-6* knockdown specifically in astrocytes, the time to onset of clinical signs of EAE is delayed in females, who also show a reduction in areas of demyelination and a decrease in cellular infiltration in the parenchyma. However, these differences in clinical score between astrocytic *Il-6*-deficient females and control littermates remain minimal compared with the clinical effects obtained with the total *Il-6* knockdown. This may be explained by the fact that astrocytic knockdown is compensated for, by IL-6 production by other cell types notably B lymphocytes. In males, on the other hand, no difference is observed, either clinically or histologically (84).

Interleukin-15 (IL-15) is poorly expressed at a basal level by astrocytes. However, Saikali’s team has demonstrated astrocytic overexpression of this cytokine in and around cortical lesions in MS patients. In their work, this team demonstrates that cluster of differentiation 8 (CD8) + T lymphocytes are located in areas where IL-15 is overexpressed, suggesting a role for astrocytic IL-15 in promoting the cytotoxic functions of CD8 T lymphocytes (85). Very few other groups have explored the role of astrocytic IL-15 in EAE or MS to our knowledge.

Interleukin-17 (IL-17) is produced in large quantities by T lymphocytes after induction of EAE. Astrocytes express the IL-17 receptor constitutively and overexpress it in neuro-inflammatory conditions (86). *In vitro*, treatment of astrocyte cultures with a cocktail of IL-17A and TNFα induces nuclear translocation of NF-κB and production of interleukins (IL-6 and IL-8) and chemokines (CXCL1, CXCL2 and CCL20) (87). The IL-17 receptor is a heterodimeric complex composed of SEFIR (after SEFs and IL-17 receptor) family proteins: interleukin 17 receptor A (IL-17RA) and interleukin 17 receptor C (IL-17RC) (88). Act 1 adaptor protein (ACT1) is a cytoplasmic protein that also belongs to the SEFIR family of proteins. Following stimulation by IL-17A, ACT1 is recruited to the IL-17R receptor via a SEFIR domain-dependent interaction. ACT1, in turn, induces the recruitment of TNF receptor associated factor (TRAF) family of kinases such as TRAF6, which activates the NF-κB pathway (89). *In vivo*, using a mouse *Act1* knockdown under the control of the Nestin promoter to induce *Act1* inhibition specifically in neuronal precursors, Kang et al. demonstrated, in deficient mice, a delay in the onset of clinical signs after EAE induction and a decrease in physical disability at acme. A decreased infiltration of inflammatory cells in the spinal cord of *Act1*-deficient mice is also observed, associated with less demyelination (90).

Interleukin-33 (IL-33) is overexpressed in the CNS of MS patients both in cortical lesions (plaques) and in healthy regions of the brain and spinal cord, compared with healthy volunteers (91). In an animal model of EAE, Chen et al. demonstrated that IL-33 is released by activated astrocytes and dying neurons during the progression of the disease and prevents EAE exacerbation. After administration of a blocking antibody directed against IL-33 in the cerebral ventricles, the authors observed an increased in the clinical scores, an increased demyelination and more CD3+ cells infiltration in the CNS (92). In 2019, the same team repeated the experiment in a mouse model with total *Il-33* knockdown. The authors confirmed the data obtained with the antibody and showed an exacerbation of the disability and spinal cord demyelination, correlated with a decrease in regulatory T cell in spleen and lymph nodes and a decrease in TH2 cell populations in the CNS of EAE-induced *Il-33* knockdown mice (93). However, these data are open to criticism since the effect observed on the pathophysiology of EAE is linked to the total inhibition of IL-33 in mice via the use of a blocking antibody or by a total *Il-33* knockdown. Thus, the specific effect of astrocytic IL-33 cannot be identified with certainty in these models. Furthermore, clinical scores at plateau phase in control animals are abnormally low for EAE models.

###### 2.2.2.3.2 Chemokines

C-C motif chemokine ligand 2 (CCL2) is the most extensively studied chemokine in MS and EAE. In EAE, the CNS cells that predominantly produce CCL2 are astrocytes and endothelial cells (94). In astrocytes, its production is dependent on *NF-κB* which, following stimulation with TNFα, is recruited to the CCL2 promoter and acts as a transcription factor (95). Using astrocytic *Ccl2* knockdown, Kim et al. showed that astrocytic CCL2 is deleterious in EAE and responsible for macrophage and T cell infiltration into the parenchyma (96). Paul et al. compared EAE-induced *Ccl2* knockdown mice at astrocytic (ACKO) and endothelial (ECKO) levels, and analyzed the pathophysiology of the disease at D9 and D16 post-induction, further determining the action of this chemokine in disrupting junctional proteins and BBB permeability. At D9 post-induction, i.e. before the appearance of the first symptoms of the disease, CLDN5 expression is reduced in control and ECKO mice, but preserved in ACKO mice, underlining the early effect of astrocytic CCL2 on the destabilization of junctional proteins. Astrocytic CCL2 is also responsible for the passage of leukocytes into the parenchyma, a perivascular cell aggregate being found at D16 in ACKO mice in contrast to ECKO and control mice. Endothelial CCL2, on the other hand, appears to participate solely in the transendothelial passage of immune cells (97).

C-C motif chemokine ligand 20 (CCL20), originally called macrophage inflammatory protein (MIP)-3a, is the ligand for the CC-chemokine receptor (CCR) 6. This receptor is located on memory T lymphocytes (98), mature B lymphocytes (99) and dendritic cells (100). Astrocytes have been identified as the main source of CCL20 in EAE. Ambrosini’s team demonstrated that this production is dependent on IL-1β and TNFα *in vivo* (101). Meares et al. also highlighted the role of IL-6 and IL-17 in transcriptional activation of the gene encoding CCL20 via phosphorylation of STAT3 and NF-κB (102). Finally, Ambrosini et al. demonstrated the chemoattractant role of astrocytic CCL20 on TH1 and TH2 lymphocytes *in vitro* using conditioned medium of IL-1β/TNFα-stimulated astrocytes (101). To date, no transgenic mice for CCL20 have been developed, making it difficult to transpose these results *in vivo*. However, *CCL20 receptor* knockdown does exist; in this mouse model, Reboldi et al. described the resistance of the knockout mice to the development of paralysis after induction of EAE. Indeed, TH1 and TH17 lymphocytes reactive against myelin were found in these animals in the spleen but not in the CNS. This is explained by the fact that TH17 lymphocytes are trapped between endothelial cells and choroid plexus epithelial cells expressing CCL20, underlining the importance of CCL20 for the passage of TH17 lymphocytes through the choroid plexus (103).

CXC motif chemokine ligand 10 (CXCL10), also known as IP-10, is a cytokine of the CXC family whose pro-inflammatory effects on tissue infiltration were first demonstrated in hepatitis C. CXC motif chemokine ligand 9 (CXCL9), or monokine induced by gamma interferon (MIG), is a chemokine of the same family whose effects have been described in tumor models. These two proteins share a common receptor, CXCR3. Simpson et al, in a post-mortem analysis of MS patient brains, described astrocytic and macrophagic expression of CXCL10 and CXCL9 at the edge of active demyelinating lesions. CXCR3 is expressed by lymphocytes in the lesion. In parallel, the authors also showed that IFNγ, known to increase CXCL10 and CXCL9 expression, is expressed in perivascular infiltrates. However, CXCL10 and CXCL9 expression is not found in chronic non-active lesions. In view of these results, the authors propose the following activation sequence: IFNγ expression by perivascular lymphocytes induces CXCL10 and CXCL9 expression by astrocytes, which in turn triggers the recruitment of CXCR3 receptor-expressing lymphocytes to the lesion (104). In addition to their role in lymphocyte recruitment, the chemokines CXCL10 and CXCL9 are also thought to play a role in astrocyte-astrocyte and astrocyte-microglia communication. Indeed, like Simpson’s team, Tanuma et al. demonstrated astrocytic and microglial expression of CXCR3 in active MS lesions. The authors hypothesize that communication between a chemokine (CXCL9/10) and its receptor CXCR3 leads to the recruitment of astrocytes and microglia to the lesions (105). In a mouse model of astrocytic knockdown for *Cxcl10 (Cxcl10^ACKO^* mice), subjected to EAE, Mills Ko et al. confirmed the deleterious role of astrocytic CXCL10 on acute disease progression via an improvement in the clinical score and a reduction in the demyelination in *Cxcl10^ACKO^* mice, with no effect on the chronic axonal loss. Regarding the lymphocyte infiltrate, no changes were observed in the parenchyma, but two major differences were highlighted by the authors: a decrease in the TH1/TH17 ratio and a decrease in the CD4+ T lymphocyte accumulation in the perivascular spaces (106).

CXC motif chemokine ligand 12 (CXCL12), also known as stromal cell-derived factor 1, is a known inhibitor of lymphocyte entry into the CNS. It is constitutively expressed by various CNS cells at low noise and overexpressed by astrocytes in MS patients. By injecting EAE-induced mice with a neutralizing anti-CXCL12 antibody, Meiron et al. demonstrated an anti-inflammatory effect of CXCL12. In addition to attracting T lymphocytes, CXCL12 also has an impact on their polarization. Treatment of mice with CXCL12 results in a switch from pro-inflammatory TH1 cells to IL-10-producing anti-inflammatory regulatory T cells (107). Furthermore, Patel et al. demonstrated that astrocytic CXCL12, by binding to its receptor CXCR4 in oligodendrocytes, promotes olidogendrocyte proliferation, migration and differentiation (80).

###### 2.2.2.3.3 Chemokine and cytokine production and regulation

The production of a large number of molecules by astrocytes under neuro-inflammatory conditions is dependent on NF-κB. Activation of the canonical or classical NF-κB pathway results in the expression of genes encoding pro-inflammatory cytokines (such as TNFα, IL-1β and IL-6), growth factors (such as granulocyte-macrophage colony-stimulating factor (GM CSF)), chemokines (IL-8, CXCL1, CXCL2, CCL2, CCL3 and CCL5), matrix metalloproteinases (MMPs) such as MMP9, pro-proliferative proteins (such as cyclin D1 and cellular myelocytomatosis oncogene (MYC)), anti-apoptotic proteins (such as B-cell lymphoma-extra-large (BCL-XL), B-cell lymphoma2 (BCL2) and FLICE-inhibitory protein (FLIP)), pro-inflammatory enzymes (such as cyclooxygenase 2 (COX2)) and inducible nitric oxide synthase (iNOS)), angiogenic factors (such as VEGFA), adhesion molecules (such as VCAM1, ICAM1 and E-selectin) and inhibitors of NF-κB signaling (such as nuclear factor of kappa light polypeptide gene enhancer in B-cells inhibitor, alpha (IκBα) and tumor necrosis factor, alpha-induced *protein* 3 (A20)).

To better characterize the effects of astrocytic NF-κB, Brambilla’s team created a transgenic mouse model of *NF-κB* inactivation in astrocytes by overexpressing, under the *Gfap* promoter, *dn-IκBα*, a NF-κB super repressor. By subjecting these mice to EAE, the authors demonstrated that inhibition of astrocytic NF-κB leads to an improvement in the mice’s clinical score, a reduction in leukocyte infiltration and a decrease in astrogliosis. These clinical and histological improvements correlate with a reduction in the expression of pro-inflammatory molecules 17 days after EAE induction (cytokines (TNFα, IFNγ, IL-1β), chemokines (CCL2, CCL5, CXCL9, CXCL10) and their receptors (CXCR2 and CCR2) (108).

#### 2.2.3 What about the NOTCH signaling in astrogliosis mechanisms?

##### 2.2.3.1 The NOTCH pathway

The *NOTCH* gene was first described in Drosophila in the 1910s, and has subsequently been found in most vertebrates, reflecting the age and conservation of this pathway (109).

While a haplo-insufficiency in Drosophila caused a notch-like anatomical defect in the wings, giving the pathway its name, the total knockout was lethal.

**Figure 5:**
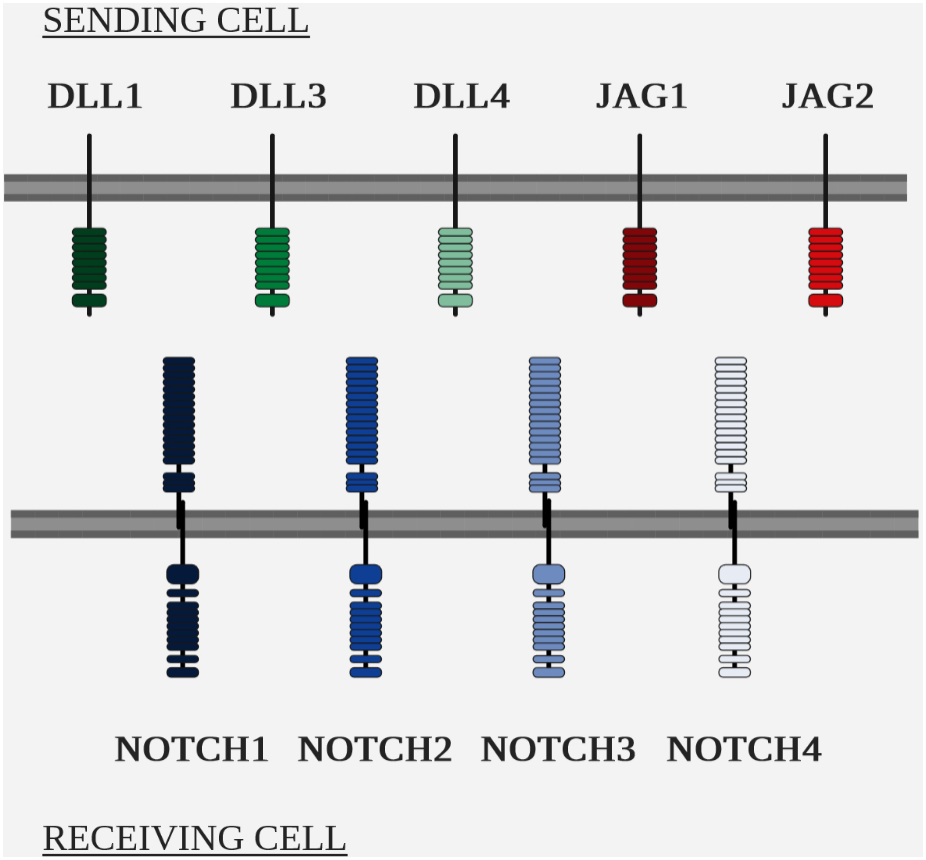
Overview of the NOTCH ligands and NOTCH receptors in mammals.

The two main players in the NOTCH pathway are the NOTCH receptors and their ligands, of the Delta/Serrate/Lag-2 (DSL) family (**Figure 5** Overview of the NOTCH ligands and NOTCH receptors in mammals).

Unlike many cellular pathways, the NOTCH pathway has no intermediary between its membrane receptor and the nuclear effector. Indeed, the intracellular part of the receptor is translocated to the nucleus after several enzymatic cleavages (110).

###### 2.2.3.1.1 Receptors

The NOTCH pathway is highly conserved within metazoans, but the number of homologous genes varies between species, e.g. Drosophila has a single *NOTCH* gene, whereas mammals have 4 paralogous *NOTCH* genes, numbered 1 to 4 and located in humans on chromosomes 9, 1, 19 and 6 respectively (111–113).

Receptors are composed of an extracellular part, a transmembrane part and an intracellular part. The extracellular part contains a variable number of tandem repeats of the endothelial growth factor (EGF) like domain. The intracellular part contains a sequence, called RBPJ association module (RAM), responsible for binding to the complex core binding factor 1 (CBF1)/suppressor of hairless/longevity assurance gene (LAG1) also named CSL or RBPJ) (114), an ubiquitous transcription factor that causes the recruitment of other transcription factors (115).

With regard to cells derived from neural progenitors, most studies on the NOTCH pathway have been carried out during development or under pathological conditions, and only very few studies have focused on the NOTCH pathway under physiological conditions in adults. NOTCH1 is the most studied in the CNS, being expressed by pyramidal neurons and playing a role in memory processes and synaptic plasticity (116, 117). However, NOTCH receptors have also been identified in adults at the BBB, in endothelial cells for NOTCH1 (118) and NOTCH4 (8), and in mural cells (pericytes and smooth muscle cells) for NOTCH3 (11).

###### 2.2.3.1.2 Ligands

As with receptors, the number of homologous genes varies for ligands. In Drosophila, two *NOTCH* ligands have been identified, *DELTA* and *SERRATE* (119), while to date, five ligands have been identified in humans: *delta like canonical notch ligand 1 (DLL1), ligand 3 (DLL3), ligand 4 (DLL4), jagged canonical notch ligand 1 (JAG1) and ligand 2 (JAG2).* They have a transmembrane structure similar to that of the receptors NOTCH (110).

###### 2.2.3.1.3 Ligand-receptor interaction

In Drosophila, the activity of the DELTA and SERRATE ligands is regulated by FRINGE, a glycosyltransferase. Its expression in the same cell as the receptor causes glycosylation of the EGF sequence repeated on the extracellular domain of the receptor, thus promoting DELTA binding and inhibiting SERRATE binding. Similarly in mammals, the FRINGE homologues, lunatic fringe (LFNG), manic fringe (MFNG) and radical fringe (RFNG), modulate the interaction of ligands with the receptor: LFNG and MFNG favor DLL1 binding and inhibit JAG1 binding, while RFNG favors DLL4 and JAG1 binding (120, 121).

Despite the differences in binding attributable to glycosylation of the NOTCH receptor, the ligands possess different intrinsic affinities for the receptors: DLL1 has a greater affinity for NOTCH2 and DLL4 for NOTCH1 (122, 123).

Cis-inhibition: when expressed on the same cell as the receptor, NOTCH ligands can inhibit these same receptors. This phenomenon is called cis-inhibition, as opposed to trans-activation, i.e. the activation of a receptor on cell A by a ligand on cell B (124). The only known functions of DLL3 to date are, for example, via cis-inhibition of NOTCH1 (125).

As with trans-activation, cis-inhibition is modulated by NOTCH glycosylation. MFNG and LFNG promote cis-inhibition of NOTCH by DLL ligands and restrict cis-inhibition by JAG ligands (126).

**Figure 6:**
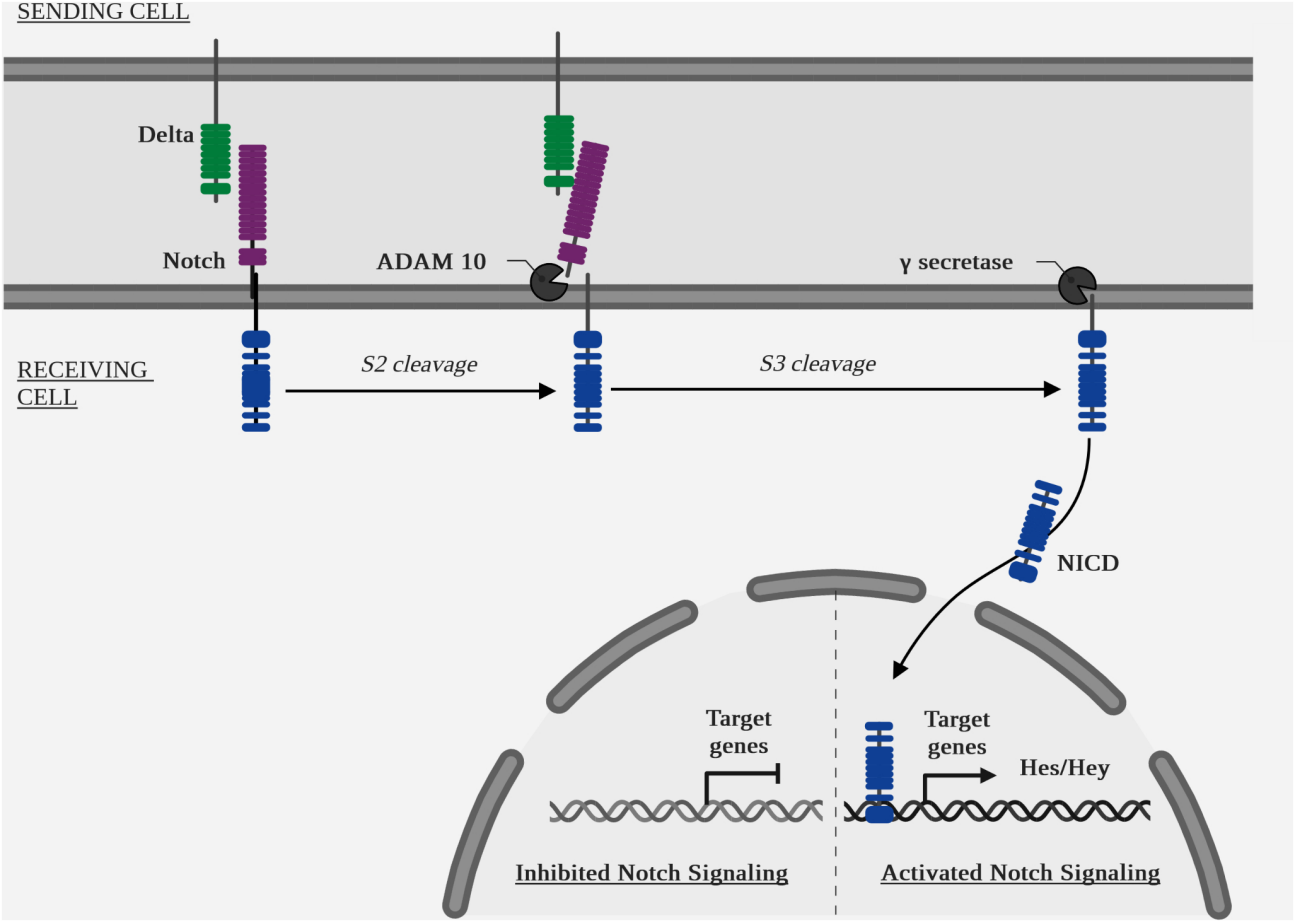
Diagram of the NOTCH pathway cascade.

Canonical pathway (**Figure 6** Diagram of the NOTCH pathway cascade): ligand binding to a receptor initiates its endocytosis, which causes a conformational change in the receptor, opening up an enzymatic cleavage site known as S2, targeted by the two isoform enzymes ADAM metallopeptidase domain 10 (ADAM10) and ADAM metallopeptidase domain 17 (ADAM17) (127). A second intracellular enzymatic cleavage at the S3 site by a γ-secretase, the protein complex containing presenilin 1 (PSEN1) and presenilin 2 (PSEN2) (128), aph-1 homolog A, γ-secretase subunit (APH1A) and aph-1 homolog B, γ-secretase subunit (APH1B), presenilin enhancer γ-secretase subunit (PSENEN) and nicastrin (NCSTN) (129), will cause translocation of the intracellular part of the receptor, NOTCH intracellular domain (NICD), to the nucleus. The target genes of the canonical NOTCH pathway are: *hes family bhlh transcription factor (HES) and hes related family bhlh transcription factor with yrpw (HEY)* (7).

The quantitative increase of NICD by cleavage and of *HES* and *HEY* by transcription is a signal of activation of this pathway.

Non-canonical pathway: there is also a NOTCH non-canonical pathway represented by several mechanisms. Mature NOTCH receptors are recycled after endocytosis (130). They can then be re-addressed to the membrane, destroyed in lysosomes or activated in endosomes, independently of a ligand. Indeed, endosomes contain ADAM enzymes and γ-secretases (131).

NICD can also interact independently of CSL, via direct interaction with other proteins, such as NF-κB (132).

##### 2.2.3.2 NOTCH1 is a positive effector of astrogliosis

In normal CNS conditions, the NOTCH pathway is only slightly active and NICD is found mainly in the cytoplasm of neurons. However, in several models of neuro-inflammation, NOTCH1 is strongly upregulated (**Table 3** Summary table of the NOTCH pathway activity *in vitro* under pathological condition in cultured astrocytes and **Table 4** Summary table of the NOTCH pathway activity *in vivo* under various neuro-inflammatory conditions).

**Table 3:** Summary table of the NOTCH pathway activity *in vitro* under pathological condition in cultured astrocytes.

*In vitro*				
Species	Pathological condition	Treatment	Effect on NOTCH pathway activity	Reference
Primary astrocytes from rat	Hypoxia	Gas mixture of 3% oxygen, 5% carbon dioxide and 92% nitrogen	Increased production of NICD	(133)
Primary astrocytes from rat	Oxidative stress	Hydrogen peroxyde 100 µmol.L^-1^ for 6 hours	Increased production of NICD	(134)
Primary astrocytes from mice	Inflammation	LPS 500 ng.mL^-1^ for 24 hours	Decreased production of NICD	(135)

**Table 4:** Summary table of the NOTCH pathway activity *in vivo* under various neuro-inflammatory conditions.

*In vivo*			
Disease	Model	Effects on NOTCH pathway in the CNS	Reference
ALS	SOD1-G93A (transgenic mouse model)	NOTCH1, JAG1, HEY1, HES1 and MAML1 are all increased in whole spinal cord	(136)
ALS	SOD1-G93A (transgenic mouse model)	NICD is increased in astrocytes	(137)
ALS	SOD1-G93A (transgenic mouse model)	NICD/JAG1/DLL4 are increased in astrocytes	(138)
ALS	SOD1-G93A (transgenic mouse model)	NICD is increased in astrocytes	(15)
ALS	Human: ALS patient post-mortem tissue	NICD is prominently present in reactive astrocytes	(15)
ALS	Human: ALS patient post-mortem tissue	NICD is decreased in the hippocampus	(139)
Stroke	MCAO (pathological mouse model)	NICD is increased in whole brain	(140)
Stroke	MCAO (pathological mouse model)	NICD is increased in astrocytes	(14)
Stroke	MCAO (pathological mouse model)	NICD is decreased in astrocytes	(141)
Traumatic injury	SCI (pathological mouse model)	NICD is markedly enhanced in A1 astrocytes at the lesion area compared to astrocytes in uninjured areas	(142)
SMA	SMNΔ7 (transgenic mouse model)	NICD is increased in astrocytes	(143)
MS	EAE (pathological mouse model)	NICD is increased in whole spinal cord	(17)

###### 2.2.3.2.1 Amyotrophic lateral sclerosis

Amyotrophic lateral sclerosis (ALS) is a neurodegenerative disease affecting motor neurons in the brain and spinal cord. Numerous teams have studied the NOTCH pathway in experimental animal models of ALS, with sometimes contradictory results.

In a mouse model of ALS, SOD1-G93A (mSOD), Wang et al. demonstrated increased activation of the NOTCH pathway in mouse spinal cords. Treatment of the mice with lithium and valproic acid, two neuroprotective drugs used in ALS patients, inhibits activation of the NOTCH pathway, the dual therapy being synergistic. However, in this study, activation of the NOTCH pathway was measured on whole spinal cord and was not studied specifically in the different cell types. Meanwhile, *in vitro* analyses were carried out only on a hybrid motor neuron/medulloblastoma cell line (136).

Two teams subsequently attempted to map the expression of the NOTCH ligands and target genes in the spinal cord. Ma et al. demonstrated a modification of the NOTCH pathway activation in mSOD mice after the onset of the disease. In these mice, the disease manifests itself around 90 to 120 days postnatal. The authors demonstrated that before this period, at day 60 for example, the NOTCH pathway is activated in motor neurons (co-localization of NICD and NeuN) in both mSOD and control mice. In 117-day-old mSOD mice, activation of the pathway is decreased in motor neurons and increased in astrocytes. In control mice, NOTCH pathway activation is absent in astrocytes (137). Liu’s team confirmed these results in the same mouse model and correlated the increase in NOTCH pathway activation in astrocytes with an increase in the expression of *HES1*, a target gene of the NOTCH pathway in these same cells (138). Conversely, in the same ALS model, Nonneman’s team found an increase expression of NICD and JAG1 in astrocytes. Indeed, a mouse model of *Jag1* inhibition specifically in astrocytes causes an increase in disease severity and a decrease in motor neuron viability as well as an increase in NOTCH pathway activation in astrocytes suggesting a beneficial role for NOTCH pathway activation in ALS (15).

Moreover, Gomez-Pinedo et al. performed immunostaining on hippocampal sections from ALS patients. The authors observed an increase in NOTCH1 expression but a decrease in NICD production associated with a decrease in the expression of γ-secretase, ADAM10 and ADAM17. The authors proposed that this decrease in NICD corresponds to a reduced activation of the NOTCH pathway in neurons. However, no quantification of neuronal or astroglial NICD was performed (139).

###### 2.2.3.2.2 Stroke

The reference murine model for the study of stroke is the Middle Cerebral Artery Occlusion (MCAO). The basic technique involves introducing a filament via the carotid artery and advancing it until it blocks the origin of the middle cerebral artery. By varying the duration of the occlusion, this model can produce transient ischemia, ischemia reperfusion, or permanent ischemia.

In a MCAO model of ischemia-reperfusion, γ-secretase activity in mouse brain increases rapidly before returning to normal. Following a one-hour ischemia, the activity is significantly higher after 2 hours of reperfusion, and its level returns to normal after 6 hours of reperfusion. NICD levels are elevated as early as one hour of ischemia and remain stable after 2 and 6 hours of reperfusion. The authors used two techniques to inhibit activation of the NOTCH pathway, antisense *Notch* transgenic mice (*NAS*) and γ-secretase inhibitors (GSI). *NAS* transgenic mice are generated by expressing an antisense *Notch1* oligonucleotide under the control of the mammary tumor virus long terminal repeat promoter. The two GSI used are: DAPT (tert-butyl(2S)-2-[[(2S)-2-[[2-(3,5-difluorophenyl)acetyl]amino]propanoyl]amino]-2-phenylacetate) and DBZ ((2S)-2-[[2-(3,5-difluorophenyl)acetyl]amino]-N-[(7S)-5-methyl-6-oxo-7H-benzo[d][1]benzazepin-7-yl]propanamide). Neurological deficits and brain damage are less severe in transgenic or GSI-treated mice than in their controls. This improved perfusion recovery correlates with reduced microglia activation and lymphocyte infiltration (144). In this study, the use of *NAS* transgenic mice and GSI did not allow us to target a particular cell type. While systemic inhibition of the NOTCH pathway in several cell types appears to have a synergistic effect on the performance of mice after ischemia, it does not provide a detailed understanding of the mechanisms modulated in these different cell types. The same team subsequently demonstrated, *in vitro* and *in vivo*, that neuronal expression of NOTCH1 is responsible for activation of the apoptotic cascade in a model of cerebral ischemia (140).

Another team focused on γ-secretase cleavage products in a subpopulation of radial glial cell marker 2 (RC2)+ reactive astrocytes. After cerebral ischemia, a subpopulation of peri-lesional proliferative astrocytes (KI67+) begins to express RC2. In these astrocytes, there is an increase in NICD and the intracellular domain amyloid beta precursor protein (APP) known as APP intracellular domain (AICD). GSI treatment after cerebral ischemia do not alter the total number of reactive astrocytes (GFAP+), but reduce the number of RC2+ astrocytes and the number of proliferative reactive astrocytes (GFAP+ KI67+). In a model of conditional astrocytic deletion for *Notch1*, the authors confirmed the results obtained with GSI: decreased number of GFAP+ reactive astrocytes, decreased number of RC2+ reactive astrocytes and increased parenchymal infiltration of CD45+ cells (14).

In contrast, Magnusson et al. found a reduced activation of the NOTCH pathway in astrocytes after cerebral ischemia. In their model of MCAO-induced ischemia, occlusion was maintained for 35 minutes in mice. The authors used a mouse line with a reporter system for cells receiving a signal from the NOTCH pathway. This system is based on the generation of a chimeric NOTCH1 receptor in which the intracellular portion is replaced by GAL4-VP16 (N1-Gal4VP16) in mice that are crossed with transgenic *UAS-lacZ* reporter mice (145). Two weeks after induction of ischemia in these mice, both NICD and β-galactosidase mRNA become undetectable in the striatum of the mice but remain abundant in the brains of uninjured mice. The authors also demonstrated that, as early as day 2 post-ischemia, astrocytes in the striatum express achaete-scute family BHLH transcription factor 1 (*ASCL1*), a proneural transcription factor. Two weeks after ischemia, clusters of doublecortin (DCX)-positive neuroblasts appear. To specifically identify striatal astrocytes, the authors injected an adenovirus expressing the Cre recombinase under the *Gfap* promoter into *R26R-YFP* mice. Seven weeks after inducing ischemia in these mice, ASCL1+ and DCX+ cells also express YFP, demonstrating that astrocytes in the striatum generate neuroblasts. Finally, RBPJ deletion in striatal astrocytes from healthy mice induces a phenotype similar to a post-ischemia phenotype, namely an increase in ASCL1 and neuroblast generation. Thus, in post-ischemia, reduced activation of the NOTCH pathway in astrocytes seems to induce a neurogenic program (141).

###### 2.2.3.2.3 Traumatic injury

Following traumatic injury to the spinal cord, a population of reactive astrocytes with a toxic phenotype A1, expressing complement C3, are found in the vicinity of the injury. Qian et al. demonstrated *in vitro* that treatment of primary human astrocytes with conditioned medium of LPS-treated microglial cells induces an A1 phenotype and increases activation of the NOTCH pathway. DAPT treatment reverses the A1 phenotype of astrocytes and astrocytic secretion of inflammatory cytokines: TNFα, IL-1β and IL-6. By incubating primary neurons with the conditioned medium of these astrocytes, the authors demonstrated their neurotoxic phenotype via an increase in neuronal apoptosis. In the group of neurons incubated with DAPT-treated astrocyte conditioned medium, neuronal apoptosis is significantly reduced, underlining the deleterious nature of activation of this pathway in astrocytes. Interestingly, the authors demonstrated an interaction between the NOTCH pathway and the STAT3 pathway: STAT3 phosphorylation is increased in A1 astrocytes, which is consistent with the literature, but the authors also demonstrated that inhibition of the NOTCH pathway, via DAPT, leads to a decrease in STAT3 phosphorylation, and that there is a direct interaction between these two pathways as demonstrated by immunoprecipitation of STAT3 by NICD. The authors suggest that NICD binds to STAT3 and facilitates its phosphorylation. The NICD/phospho-STAT3 complex would then be translocated to the nucleus and act as a transcription factor. However, this hypothesis remains to be proven (142).

###### 2.2.3.2.4 Spinal muscular atrophy

Spinal muscular atrophy (SMA) is an orphan hereditary disease characterized by skeletal muscle weakness and atrophy due to decreased transcriptional levels of survival motor neuron (SMN) protein caused by mutation or deletion of the *survival motor neuron 1 (SMN1)* gene. *SMN* is encoded by 2 genes that differ mainly by one nucleotide located in exon 7. The majority of *SMN2* transcripts thus yield an SMN protein truncated at exon 7 (SMNΔ7). A commonly used mouse model of spinal muscular atrophy is the *SMNΔ7* model in which the endogenous murine gene *Smn1* is inactivated and replaced by two human genes, *SMN2* and *SMN2Δ7 (mSmn-/-; hSMN2+/+, hSMN2Δ7+/+).*

Ohuchi et al. showed an increase in the level of NICD and P-STAT3 in the spinal cord of SMNΔ7 mice at P5, associated to an increase at the level of GFAP+ astrocytes (143). Previously, using fibroblasts derived from the skin of a SMA patient, the authors generated induced pluripotent stem cells (SMA-iPSCs) and showed that differentiation into motor neurons (SMA-iPSC-MN) also results in the production of a GFAP+ astrocyte population (146). The use of LY-411575, a GSI, allows a reduction in GFAP+ astrocyte differentiation in the SMA-iPSC-MN model, highlighting the importance of the NOTCH pathway in astrocyte versus neuron differentiation. *In vivo*, intraventricular injection of the same inhibitor at P2 and P5 in *SMNΔ7* mice improves motor deficit. Pathologically, GSI-treated mice have fewer GFAP+ astrocytes in the medulla than controls (143).

###### 2.2.3.2.5 Hypoxia

Using primary rat astrocytes cultured under a gas mixture of 3% oxygen (O_2_), 5% carbon dioxide (CO_2_) and 92% nitrogen (N_2_), Zhang et al. showed that hypoxia induces activation of the NOTCH pathway at 12 hours in astrocytes correlated with an increased proliferation and production of the pro-inflammatory cytokines, TNFα and IL-1β. *In vivo*, the authors showed that rat treated with DAPT show a decreased hypoxia-induced astrocytic reactivity and less VEGFA production (133).

###### 2.2.3.2.6 Oxidative stress

Oxidative stress causes protein oxidation and is implicated in several inflammatory and degenerative neurological pathologies. Sulfiredoxin-1 (SRXN-1) is an endogenous antioxidant protein with neuroprotective effects (134). To study the effect of oxidative stress, Li et al. added hydrogen peroxide (H_2_O_2_) at a final concentration of 100µmol.L^-1^ to the culture medium of primary rat astrocytes and incubated the cells for 6 hours. Under oxidative stress, SRXN-1 expression is increased in astrocytes. The number of apoptotic astrocytes is increased in the H_2_O_2_ group, and the addition of *siSRXN-1* potentiates cell apoptosis, confirming the protective antioxidant role of SRXN1. Interestingly, the NOTCH pathway is activated in astrocytes after incubation with H_2_O_2_ and DAPT treatment and causes an increase in H_2_O_2_-induced astrocyte apoptosis. Together these results suggest a protective role for the NOTCH pathway in astrocytes following oxidative stress (134).

###### 2.2.3.2.7 Experimental autoimmune encephalomyelitis

Liu et al. investigated the role of IL-9, produced by T helper 9 (TH 9) lymphocytes, in astrogliosis. Treatment of primary murine astrocytes with IL-9 resulted in an increased activation of the NOTCH pathway and STAT3 phosphorylation, as well as increased transcritional levels of the pro-inflammatory cytokines IL-6, TNF-α, CXCL10 and CCL2. The authors also demonstrated the transcription of a 446-base-pair long non-coding RNA (*lncRNA*) identical to an antisense sequence of the NOTCH gene (*lncRNA Gm13568*) after IL-9 stimulation. *In vitro*, treatment with a lentivirus inhibiting *Gm13568* or with a small hairpin RNA (*shRNA*) directed against *NOTCH1* gave identical results, namely inhibition of the NOTCH pathway in astrocytes, leading to reduced activation of the STAT3 pathway, and inhibition of production of the pro-inflammatory cytokines IL-6, TNF-α and CXCL10. *In vivo*, in a mouse model of MOG-induced EAE, the authors showed that injection of lentiviruses inhibiting *NOTCH1* or inhibiting *Gm13568* delays disease onset and reduces clinical signs in mice, which is correlated with preservation of myelin and a reduction in immune cell infiltrates in the medullar parenchyma (17).

###### 2.2.3.2.8 Contradictory data

Acaz-Fonseca et al. on the other hand, published a study in 2019 that runs counter to the above-mentioned studies describing the deleterious effects of NOTCH pathway activation in neuro-inflammatory pathologies. Indeed, after treatment of primary murine astrocytes with LPS, the authors found an increase in *JAG1* transcriptional levels, but a decrease in NOTCH pathway activation (lower *HES-5* effector transcriptional levels and reduced NICD release). According to the authors, the decrease in the NOTCH pathway activity has an effect on astrocyte morphology, but not on the production of pro-inflammatory factors (135).

In view of all the studies analyzed above, on the role of the NOTCH pathway during neuro-inflammation, it is clear that effects of inactivation of this signaling depend on several factors: first the type of pathology studied, the timing of the knockout and/or of the observations that follows; and second, the method used for the knockout, genetic or pharmacological, and the target, specific, i.e. ligand or receptor, or non-specific, e.g. GSI.

## 3 Discussion

A complex network of intercellular signaling occurs between cells of the NVU during neuro-inflammation (1, 147). Specifically, astrocytes acquire a reactive phenotype, named astrogliosis, defined by morphological and molecular changes that drive BBB breakdown, acute inflammatory injury as well as chronic neurodegeneration. Understanding the pathways controlling reactive astrogliosis and BBB function is of considerable translational interest to the field of neuro-immunology.

In this review, we first present the state of the art on reactive astrogliosis under neuro-inflammatory conditions, particularly in MS. We then focus on the NOTCH signaling involvement in astrogliosis. Interestingly, the very detailed consensus statement published by C. Escartin et al. in 2021, which lists all the reactive astrocyte markers fully identified in the literature (148), doesn’t cover the NOTCH signaling. However, a wealth of publications agrees on the fact that NOTCH1 is a central regulator of astrogliosis in several models of neuro-inflammation and is involved in the pathophysiology of various diseases, notably MS and EAE.

While the role of the NOTCH1 receptor in reactive astrocytes is well documented, that of its ligands is much less so. For example, DLL4 ligand expression has only been reported once, anecdotally, in reactive astrocytes, following brain injury (149). The important role of DLL4-NOTCH1 signaling in the cardiovascular system is already widely appreciated, but little is known about this specific pathway in reactive astrogliosis during neuro-inflammation. Thus, more investigations will be needed to explore the DLL4-NOTCH1 interaction in reactive astrocytes under neuro-inflammatory condition such as MS.

To further investigate the role of the NOTCH pathway in neuro-inflammation, we could also ask whether there is an interaction, through the NOTCH signaling, between reactive astrocytes and other cells from the NVU notably endothelial cells and pericytes which express actors of the NOTCH pathway (8–10, 150).

Moreover, literature shows that immune cells also express NOTCH signaling markers and it is well known that during MS and EAE, immune cells penetrate the BBB and then accumulate within the perivascular space in contact with the astrocytic endfeet (2, 3). Therefore, as NOTCH signaling can influence the differentiation and function of T lymphocytes (150, 151) and because novel emerging properties of the DLL4-NOTCH signaling pathway have recently been identified in pathology, notably in controlling the CD4+/CD8+, Th17/Treg balance both in experimental autoimmune uveitis and EAE (152, 153), studying the NOTCH pathway between astrocytic endfeet and infiltrated inflammatory cells is of particular interest to the field of neuro-immunology.

Overall this review proves that NOTCH1 is a recognized marker of astrogliosis in neuro-inflammation and underlines the importance to take a closer look at the NOTCH signaling in reactive astrocytes, but also NVU neighboring cells, in the pathophysiology of neuro-inflammation like MS condition, in order to investigate the possibility of targeting this pathway therapeutically.

## Author contributions

PM: Writing – original draft, Writing – review & editing, Conceptualization. CC: Funding acquisition, Investigation, Supervision, Writing – original draft, Writing – review & editing.
